# Associations Between Sign Language Skills and Resting-State Functional Connectivity in Deaf Early Signers

**DOI:** 10.3389/fpsyg.2022.738866

**Published:** 2022-03-18

**Authors:** Emil Holmer, Krister Schönström, Josefine Andin

**Affiliations:** ^1^Linnaeus Centre HEAD, Swedish Institute for Disability Research, Department of Behavioural Sciences and Learning, Linköping University, Linköping, Sweden; ^2^Center for Medical Image Science and Visualization, Linköping, Sweden; ^3^Department of Linguistics, Stockholm University, Stockholm, Sweden

**Keywords:** sign language, resting-state functional connectivity, deafness, brain-behavior association, fMRI

## Abstract

The processing of a language involves a neural language network including temporal, parietal, and frontal cortical regions. This applies to spoken as well as signed languages. Previous research suggests that spoken language proficiency is associated with resting-state functional connectivity (rsFC) between language regions and other regions of the brain. Given the similarities in neural activation for spoken and signed languages, rsFC-behavior associations should also exist for sign language tasks. In this study, we explored the associations between rsFC and two types of linguistic skills in sign language: phonological processing skill and accuracy in elicited sentence production. Fifteen adult, deaf early signers were enrolled in a resting-state functional magnetic resonance imaging (fMRI) study. In addition to fMRI data, behavioral tests of sign language phonological processing and sentence reproduction were administered. Using seed-to-voxel connectivity analysis, we investigated associations between behavioral proficiency and rsFC from language-relevant nodes: bilateral inferior frontal gyrus (IFG) and posterior superior temporal gyrus (STG). Results showed that worse sentence processing skill was associated with stronger positive rsFC between the left IFG and left sensorimotor regions. Further, sign language phonological processing skill was associated with positive rsFC from right IFG to middle frontal gyrus/frontal pole although this association could possibly be explained by domain-general cognitive functions. Our findings suggest a possible connection between rsFC and developmental language outcomes in deaf individuals.

## Introduction

Sign languages are the primary mode of communication in Deaf communities across the world. Similar to spoken languages, signed languages have syntactical, lexical, and sub-lexical structures that differ between languages across geographical regions (Mathur and Rathmann, 2014). However, sign language is expressed in the manual-visual domain, whereas speech is formed in the oral-aural modality. In spite of the modality differences, the existing evidence suggests that neurobiological correlates of language processing overlap to a great deal across modalities (MacSweeney et al., 2008; Malaia and Wilbur, 2010; Cardin et al., 2020a; Trettenbrein et al., 2021). Most previous studies have applied task-based functional magnetic resonance imaging (fMRI) to identify brain regions that are associated with the processing of sign language. These studies have improved our understanding of neural structures involved in sign language perception and understanding. However, functional connectivity can advance our understanding of how different language-relevant brain regions work together for optimal language processing (Fedorenko and Thompson-Schill, 2014; Hagoort, 2019). In the present study, we investigate associations between sign language proficiency and resting-state functional connectivity (rsFC) in deaf early signers. Thus, we explore whether individual differences in sign language skills are associated with how brain regions are intrinsically and functionally related.

Hickok and Poeppel (2007) proposed a neural model of language processing with core language regions in the superior temporal cortex bilaterally, and dorsal and ventral processing streams representing different functional operations. The dorsal stream is left-hemisphere biased and includes the parieto-temporal intersection region, as well as premotor and inferior frontal cortical nodes. The ventral stream, on the other hand, covers bilateral posterior middle and inferior temporal cortical regions, and the left anterior middle temporal gyrus and inferior temporal sulcus. The ventral stream is related to the mapping of an incoming language signal to its meaning, whereas the dorsal stream deals with production. However, it should be noted that consensus does not exist regarding the exact functions and anatomical distribution of the streams, and alternative accounts (e.g., Friederici, 2012; Bornkessel-Schlesewsky and Schlesewsky, 2013; Ullman, 2016; Hagoort, 2019) have a somewhat different emphasis than the model proposed by Hickok and Poeppel (2007). Nevertheless, models overlap with similar functions located across temporal, parietal, and frontal regions, and a dual-stream model finds support in meta-analytic work on speech processing (Adank, 2012; Walenski et al., 2019).

Several studies have reported shared neural activation across spoken and signed language (Söderfeldt et al., 1994b,^1997^; Petitto et al., 2000; MacSweeney et al., 2002; Sakai et al., 2005; Emmorey et al., 2007, 2014; Courtin et al., 2011; Zou et al., 2012; Johnson et al., 2018; Moreno et al., 2018; Finkl et al., 2020), although a recent study by Evans et al. (2019) indicated that only semantic, not form-based, representations share neural activation patterns. Due to modality-specific operations needed for processing of manual-visual language, some suggest that certain brain regions (e.g., the superior parietal lobule) might be specifically engaged for sign language (Söderfeldt et al., 1994a; Zou et al., 2012; Emmorey et al., 2014). It has also been suggested that the involvement of the right hemisphere might be more prominent for sign language than for speech (Newman et al., 2002, 2010; Emmorey et al., 2005, 2014; Sakai et al., 2005). The role of the right hemisphere has further been proposed to be dependent on proficiency (Malaia and Wilbur, 2010) and the age of acquisition (AoA) of sign language (Neville et al., 1997; Newman et al., 2002; Mayberry et al., 2011). In addition, Stroh et al. (2019) suggested that deaf signers compared to hearing signers might recruit regions in the right hemisphere to a greater degree for certain linguistic tasks.

In a recent meta-analysis of the neural underpinnings of sign language processing, Trettenbrein et al. (2021) proposed that the right inferior frontal gyrus (IFG) and right middle temporal gyrus are specifically recruited for sign language processing. These two regions are typically not regarded as “language regions”, but based on a recent meta-analysis by Walenski et al. (2019), temporal lobule activation for spoken language processing was described as bilateral, in line with the dual-stream model proposed by Hickok and Poeppel (2007). Trettenbrein et al. (2021) further identified critical nodes for sign language processing in the left IFG and precentral/middle frontal gyrus, corroborating reports from spoken language (Walenski et al., 2019). Trettenbrein et al. (2021) also noted that previous literature indicates a role of left middle gyrus, superior temporal gyrus (STG), supramarginal gyrus, and bilateral superior parietal lobules in sign language processing. These regions did show an effect of sign language processing when compared to rest conditions, but the activation overlapped with activation from non-linguistic sign-like actions from an independent set of studies. Thus, these regions might not be critically involved in linguistic aspects of sign language processing although the studies were not designed to test this directly. Due to the limited number of studies, Trettenbrein et al. (2021) could not differentiate between regions that might be specific for production versus comprehension, or syntactical, lexical, and phonological levels of processing. It thus remains unclear how different regions relate to the type of linguistic processing in sign language. Some propose that the left supramarginal gyrus is particularly important for phonological analysis in sign language (Corina et al., 1999; Emmorey et al., 2003; MacSweeney et al., 2008), and activation of the inferior frontal and superior and middle temporal cortices is associated with sentence level processing of speech (Walenski et al., 2019). In addition, language comprehension seems to be bilaterally distributed to a larger degree than language production (Walenski et al., 2019).

The literature indicates that sign language proficiency might influence neural responses. For example, AoA, which likely influences proficiency (Twomey et al., 2017), seems to produce lateralization effects (e.g., Newman et al., 2002; Mayberry et al., 2011). Newman et al. (2002) compared neural activation for real American Sign Language sentences compared to sentence-like pseudo-sign utterances, and reported a right hemisphere effect of AoA, with activation of angular gyrus in early, but not late, learners. AoA effects have further been reported to be associated with the level of activation in other regions. Mayberry et al. (2011) regressed neural activation on AoA and showed that, with earlier AoA, there was stronger activation in bilateral dorsolateral prefrontal cortex, left anterior insula/frontal operculum, left IFG, left ventral premotor region, and bilateral STG. Later AoA, on the other hand, was associated with stronger activation of left lingual gyrus and left middle occipital gyrus. Twomey et al. (2020) reported that late as compared to early signers, regardless of hearing status, had stronger activity in the occipital segment of the left intraparietal sulcus. Further, early deaf signers showed greater activation in the left posterior superior temporal cortex in response to real sign language sentences as compared to made-up signs. In yet another study, Cheng et al. (2019) reported that language deprivation in three cases of deaf individuals was associated with altered structural connectivity in the left dorsal arcuate fasciculus pathway, a fiber tract connecting superior temporal to frontal regions. Thus, early access to sign language seems to produce effects on neural activation in occipital, parietal, temporal, and frontal regions, but also influences the development of language-relevant structural pathways. It is likely that level of proficiency is associated with differences in neural activation patterns. Emmorey et al. (2015) reported that the regional neural activation that correlates with behavioral performance on linguistic tasks differs depending on the specific linguistic operation of the task. For example, they saw that fingerspelling ability was negatively associated with neural activation in right frontal regions, whereas sentence processing of sign language was negatively associated with activation in angular gyrus and middle temporal gyrus.

Apart from the above-described association between sign language skills and region-specific activations, associations between language skills and brain activity have also been investigated using rsFC. For example, Qian et al. (2016) saw associations between reading skills and rsFC of visual dorsal stream regions (i.e., involved in spatial processing) and regions invoked by reading tasks, such as the fusiform gyrus. In another study, Koyama et al. (2011) reported an association between reading skills and rsFC between the left fusiform gyrus and left frontal and inferior parietal regions. In addition, Chai et al. (2016) investigated associations between second language acquisition and rsFC based on two regions of interest, the anterior insula/frontal operculum and the visual word form area in the fusiform gyrus. They reported positive associations between degree of second language acquisition and connectivity between anterior insula/frontal operculum and left posterior STG as well as the dorsal anterior cingulate cortex. In a study on associations between rsFC and language skills in deaf adults, Li et al. (2013) investigated rsFC within and between superior temporal regions and how connectivity was related to written language skills in congenitally deaf adults, adults with acquired deafness, and hearing adults with no knowledge of sign language. Connectivity between the middle and anterior parts of the superior temporal cortex was associated with written language skills in participants with deafness, but not in hearing participants. However, Li et al. (2013) did not report any associations between written language skills and rsFC outside superior temporal regions. In summary, there are studies showing associations between spoken language skills and rsFC in both hearing (Chai et al., 2016) and deaf (Li et al., 2013) individuals, but as far as we know, associations between sign language proficiency and rsFC have hitherto not been described in the literature.

Given the available evidence, there is a multitude of potentially relevant brain regions to include in an analysis of associations between rsFC and sign language proficiency. For the purposes of the present study, we based the selection of seeds of interest in our connectivity analysis on two of the most studied regions in relation to language processing, i.e., the IFG and STG (e.g., Trettenbrein et al., 2021). To restrict the number of statistical tests performed in our exploratory analysis, only four nodes were included as seeds: bilateral IFG and posterior STG. This selection of regions does not include all potentially relevant regions proposed by dual-stream models, such as the one by Hickok and Poeppel (2007), but it overlaps with such models. For the selected regions, we estimated seed-to-voxel based connectivity for each individual and correlated with behavioral performances. We included two separate measures of sign language proficiency, one that taps onto phonological skill and another that represents sign language sentence processing skill. This was because there is reason to believe that the type of linguistic operation might affect which specific neural networks that are involved (Hickok and Poeppel, 2007; Friederici, 2012; Bornkessel-Schlesewsky and Schlesewsky, 2013; Hagoort, 2017).

## Materials and Methods

### Participants

Participants were recruited for a larger project (see Andin et al., 2021) and 15 (out of 17) had complete data on measures of sign language phonological and sentence processing, as well as an fMRI resting-state session (mean age = 35.0, *SD* = 7.8, min 22, max 48). All were right-handed and had normal or corrected-to-normal vision. Non-verbal cognitive ability was normal or above normal, as assessed on the Visual Puzzles subtest from the Wechsler Adult Intelligence Scale, Fourth Edition (WAIS-IV, Wechsler, 2008). The Visual Puzzles subtest has one of the highest factor loadings (0.72) on the index of non-verbal ability (the Performance Index) in the Swedish version of WAIS-IV, which makes it a good proxy for non-verbal cognitive ability when time constraints limit the number of tests to include. Six participants had a university degree, and the rest had completed high school. Nine were deaf from birth and the remaining six became deaf before the age of three. Five were native signers, and the rest were exposed to sign language before the age of three. All used Swedish Sign Language (*Svenskt teckenspråk*; STS) as their primary language. The study was reviewed and approved by the regional ethical review board in Linköping (Dnr 2016/344-31). Participants gave their written informed consent and received a gift as a compensation for their participation.

### Sign Language Proficiency Measures

#### Cross-Modal Phonological Awareness Test

The Cross-modal Phonological Awareness Test (C-PhAT; Holmer et al., 2016) was used as a measure of sign language phonological awareness. Pairs of printed characters (two letters or a letter and a number) were presented and the participant had to respond whether the STS handshapes representing the two characters were the same or not, regardless of their orientation and location. Two lists of 24 pairs, eight of which overlapped in handshape (see Holmer et al., 2016), were presented in counterbalanced order across participants. The order of pairs was randomized for each participant. Stimuli were presented until the participant made a response, or for a maximum of 20 s. The interstimulus interval was 1 s. The dependent measure was average response time in ms. Reliability was estimated based on the correspondence in performance across lists, *r* = 0.75.

#### Swedish Sign Language Sentence Repetition Test

To assess sign language sentence reproduction, the Swedish Sign Language Sentence Repetition Test (STS-SRT, Schönström and Hauser, 2021), a Swedish adaptation of the American Sign Language Sentence Repetition Test (ASL-SRT; Hauser et al., 2008), was used. Filmed STS sentences (*N* = 31), of different length and difficulty, produced by a deaf native signing man, were presented to the participant. The participant watched each sentence and was instructed to reproduce it exactly as it was signed in the video. Video clips were presented on a laptop (12” screen), and approximately 8 s were left for a response before the next trial started. The front camera on the laptop was used to film responses, which were scored on a later occasion by the second author (who is a deaf native user of STS). One point was awarded for each sentence that was an exact replication of the sentence presented in the video. The dependent variable was the number of correctly reproduced sentences. As estimates of reliability, internal consistency and inter-rater reliability provided excellent values in a previous study, with Cronbach’s α = 0.92 and ICC = 0.90 (Schönström and Hauser, 2021). Furthermore, the test provides evidence for good validity as suggested by better performance in adults than in children, and that delayed language acquisition is associated with lower scores.

### Resting State Functional Connectivity

#### Data Acquisition

MR imaging was performed on a 3T scanner (Siemens Magnetom Prisma, Siemens Healthcare, GmbH) with a 64-channel head coil at the Center for Medical Image Science and Visualization (Linköping University, Sweden). Functional images were acquired during continuous scanning using a BOLD multi-plex EPI sequence during a 10-min resting-state scan with the following parameters: FOV = 192 × 192 mm, voxel size 3 × 3 × 3 mm, TR = 1,340 ms, TE = 30, FA = 69°, number of slices = 48, 440 volumes, interleaved/simultaneous acquisition. Structural images were acquired in the beginning of the session using a T1 MPRAGE 3D-sequence; FOV = 288 × 288, acquisition matrix = 208 × 288 × 288, voxel size 0.90 × 0.86 × 0.86 mm, TR = 2,300 ms, TE = 2.36 ms, TI = 900 ms, FA = 8°. Between the structural and resting-state scans, four runs of task-based fMRI were performed (see Andin et al., 2021).

#### Connectivity

Resting-state functional connectivity (rsFC) was analysed using Conn functional connectivity toolbox (version 20b, RRID:SCR_009550).^1^ For each participant, seed-to-voxel connectivity estimates were obtained by correlating the BOLD time series in selected seed regions with all other voxels in the brain. The four seeds (bilateral IFG and posterior STG) included all nodes from the language network in the network atlas defined by Conn. Data were preprocessed using the standard preprocessing pipeline in Conn, including functional realignment, unwarping and co-registration to the first scan, slice-timing correction, outlier detection by computation of framewise displacement, normalization into standard MNI space, structural segmentation into gray matter, white matter, and CSF tissue classes, and smoothing using a Gaussian kernel of 8 mm full width half maximum to reduce signal-to-noise ratio. Linear regression using the anatomical component-based noise correction (aCompCor; Chai et al., 2012) algorithm was implemented at the first level to remove confounding factors including subject-specific physiological noise from white matter and cerebrospinal areas, motion parameters, outlier scans (scrubbing), and session-related slow trends. Finally, a band-pass filter of 0.008–0.09 Hz was applied to remove high-frequency noise and low-frequency drift.

### Statistical Analysis

Descriptive statistics were calculated for the behavioral measures, i.e., C-PhAT (response time), STS-SRT (raw score), and Visual Puzzles (raw score). Further, parametric correlations were used to estimate associations between predictor variables. Statistical analyses of behavioral measures were performed in IBM SPSS Statistics (version 26). Then, associations between sign language skills and brain connectivity were estimated using the Conn toolbox (see above). For each seed region, we tested whether any statistically significant rsFC associations could be observed for either response time on C-PhAT, or number of correct responses on STS-SRT. These tests were corrected for age since variability in age has been reported to influence rsFC even in young to middle-aged adults (Xiao et al., 2018). When appropriate, we also controlled for non-verbal cognitive ability. In the second-level analyses, mean-centered predictors were entered as covariates in the seed-to-voxel analysis for each seed. Two statistical tests were conducted for each of the four seeds, one for C-PhAT and one for STS-SRT. Thus, seeds were treated as four separate groups of tests, and the *p*-value was corrected for multiple tests within each group by applying Bonferroni correction. With an α of 0.05, an association between performance on one of the behavioral tasks and rsFC was considered statistically significant with a false discovery rate (FDR) *p*-value < 0.025 at the cluster level (0.05 divided by the two tests for each seed). This liberal approach to correction for multiple tests was applied to maximize statistical power. For testing of statistically significant peaks within a statistically significant cluster, an uncorrected *p*-value of 0.001 was applied.

### Procedure

Before arriving at the laboratory, the experimenter checked if the participants adhered to the inclusion criteria based on responses in an online questionnaire. Testing started with participants being informed about the study and signing an informed consent form. Half of the participants then continued with behavioral testing and the other half with MR-scanning. Tests included screening of visual acuity and contrast sensitivity, the Visual Puzzles subtest from WAIS-IV, C-PhAT, and STS-SRT, as well as a set of cognitive tasks not reported here. Behavioral testing lasted for approximately 60 min. The MR-scanning, including a structural run, an experimental task, and the 10-min long resting-state run, lasted for 45 min. During the resting-state run, participants were instructed to focus on a white plus sign on a black background, presented virtually through MR-goggles (VisuaStim Digital, Resonance Technology, Inc.). Participants were also instructed not to fall asleep. An accredited STS interpreter was present during testing and provided verbatim translation of instructions of responses to questions. Participants communicated in STS via a video camera in the scanner.

## Results

### Performance on Behavioral Measures

For descriptive statistics on, and correlations between, behavioral measures see Table 1. One significant outlier was detected on C-PhAT, and this individual was excluded from further analyses that included this task. The association between C-PhAT and STS-SRT was not statistically significant (see Table 1), suggesting that these measures tap onto different language processes. Further, performance on C-PhAT, *r*(14) = −0.54, *p* = 0.045, but not STS-SRT, *r*(15) = −0.07, *p* = 0.82, was associated with performance on Visual Puzzles, our index of non-verbal cognitive ability. To control for the influence of non-verbal cognitive ability in associations observed between C-PhAT performance and resting-state functional connectivity (rsFC), performance on Visual Puzzles was used as a covariate in connectivity analyses involving C-PhAT.

**TABLE 1:** Descriptive statistics (means and standard deviations) on behavioral measures and their Pearson r correlations.

				Correlations	
	*N*	*M*	*SD*	STS-SRT	VP
C-PhAT	15	1.698	406	0.29	−0.54*
STS-SRT	15	17.9	4.1		−0.07
VP	15	17.8	4.6		

*C-PhAT, Cross-modal Phonological Awareness Test; STS-SRT, Swedish Sign Language Sentence Repetition Test; VP, Visual Puzzles subtest from WAIS-IV.*

*p < 0.05.

### Performance on Cross-Modal Phonological Awareness Test and Intrinsic Connectivity

For C-PhAT, connectivity from right IFG to a cluster peaking in the left middle frontal gyrus/frontal pole was negatively associated with response time, *t*(11) = 8.26, β = 0.00058, *R*^2^ = 0.87, *p* < 0.001. However, when controlling for performance on Visual Puzzles, the association was not statistically significant (*p* = 0.18 for a similar association including a smaller cluster in the left middle frontal gyrus/frontal pole). Thus, better performance (shorter response time) was associated with stronger connectivity from right IFG to left middle frontal gyrus/frontal pole when controlling for age, but not when also controlling for the influence of non-verbal cognitive ability (i.e., Visual puzzles). There were no significant associations between C-PhAT performance and rsFC from left IFG or bilateral posterior STG.

### Performance on Swedish Sign Language Sentence Repetition Test and Intrinsic Connectivity

**FIGURE 1:**
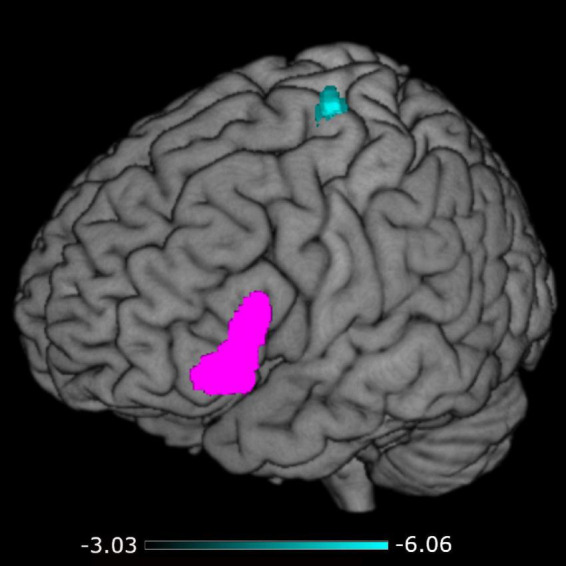
Connectivity from the left inferior frontal gyrus (seed in pink) to the cluster in sensorimotor regions (in cyan). The color map indicates the strength (as *t*-values) of connectivity within the cluster.

**FIGURE 2:**
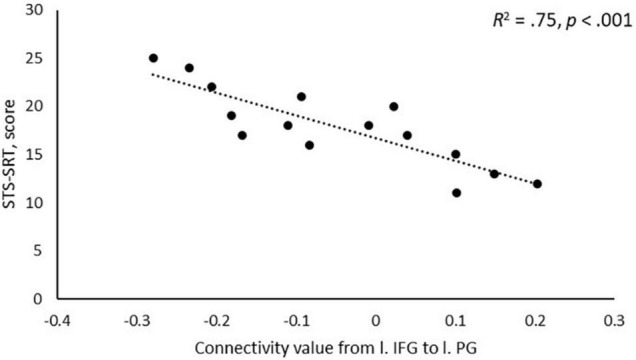
Scatterplot of the association between performance on Swedish Sign Language Sentence Repetition Task (STS-SRT; score on *y*-axis) and resting-state functional connectivity (connectivity value on x-axis) between the seed in left inferior frontal gyrus (l. IFG) and the peak in left precentral gyrus (l. PG).

A negative association was found between STS-SRT performance and rsFC from the left IFG to a cluster with a peak in the precentral gyrus, *t*(12) = 6.06, β = −0.032, *R*^2^ = 0.75, *p* < 0.001 (see Table 2). Worse sign language sentence processing skill was thus associated with stronger positive connectivity from left IFG to sensorimotor regions, after controlling for age-related differences in rsFC. The statistically significant cluster, and strength of connectivity within this cluster, is displayed in Figure 1. For a scatterplot of the association see Figure 2. There were no significant associations between STS-SRT performance and rsFC from right IFG or bilateral posterior STG.

**TABLE 2:** Statistically significant associations between Sign Language Proficiency Variables (STS-SRT and C-PhAT) and Resting-State Functional Connectivity, controlling for age.

Behavioral measure	Seed	Cluster peak location	Association	Cluster size (voxels)	Cluster size *p^a^*	Cluster peak (MNI)			Peak *t*	Peak *p^b^*	*R* ^2^
						x	y	z			
C-PhAT	r. IFG	l. MFG/FP (BA 45)	Positive	98	0.014	−40	48	18	8.26	0.000005	0.87
STS-SRT	l. IFG	l. PG (BA 6)	Negative	102	0.016	−18	−28	66	6.06	0.00006	0.75

*C-PhAT, Cross-modal Phonological Awareness Test; STS-SRT, Swedish Sign Language Sentence Repetition Test; l, left; r, right; IFG, inferior frontal gyrus; MFG, middle frontal gyrus; FP, frontal pole; PG, precentral gyrus.*

*^a^FDR-corrected (p < 0.025 is regarded as statistically significant).*

*^b^Uncorrected (p < 0.001 is regarded as statistically significant).*

## Discussion

In the present, explorative study, we investigated how individual variability in sign language proficiency, at phonological and sentence levels, is associated with resting-state functional connectivity (rsFC). More specifically, we investigated associations between two different types of sign language skills: phonological skill and sentence processing skill, and rsFC from bilateral IFG and posterior STG to the rest of the brain. Faster phonological processing was positively associated with stronger connectivity between right IFG and left middle frontal gyrus/frontal pole; however, this association did not remain after controlling for non-verbal cognitive ability. Worse sign language sentence processing ability was associated with stronger positive connectivity from left IFG to sensorimotor regions. Thus, rsFC between prefrontal and sensorimotor language regions seems to co-vary with sign language reproduction skill.

### Resting-State Functional Connectivity and Sign Language Processing

We saw that rsFC from the right IFG to left middle frontal gyrus/frontal pole is negatively associated with performance on a speeded phonological awareness task, which might suggest that the strength of communication between these regions at rest is indicative of phonological skill. However, this association was not statistically significant after control for non-verbal cognitive ability (as measured on the Visual Puzzles sub-test from WAIS-IV, Wechsler, 2008). Thus, it is possible that the association we see is driven by non-linguistic, cognitive skills. Due to the small sample in the present study, we had limited power to detect associations, and controlling for multiple covariates (i.e., age, non-verbal cognitive ability), as we did here, reduces the degrees of freedom even more. Thus, associations might exist that we could not detect. On the other hand, a small sample might produce spurious and random results that do not replicate. That is, the association we saw in the first place might have occurred by chance. In their recent meta-analysis, Trettenbrein et al. (2021) noted that the right IFG is involved in sign language processing. However, based on the available literature we see no particular reason why rsFC between this region and left middle frontal gyrus/frontal pole should be associated with phonological processing of sign language. Instead, such connectivity might reflect intrinsic activation within a lateral frontoparietal network used in the processing of executively demanding tasks (Uddin et al., 2019; Witt et al., 2021). This could possibly explain why the association we first saw disappeared when controlling for non-verbal cognitive ability.

We further saw that those who struggle more with reproducing sign language sentences correctly have stronger rsFC between left IFG and a cluster peaking in the left precentral gyrus. In the context of language processing, left IFG is typically described as a control region involved in language production and complex linguistic analysis (Hickok and Poeppel, 2007; Friederici, 2012; Bornkessel-Schlesewsky and Schlesewsky, 2013; Hagoort, 2017), and Corina et al. (1999) concluded that left IFG is critically involved in sign language production. Precentral regions have been proposed to be involved in the processing of movement of sign language (Emmorey et al., 2014). However, in recent meta-analytic work on both signed and spoken language, motor regions have been reported to be invoked also by linguistic processing (Walenski et al., 2019; Trettenbrein et al., 2021). In the dual-stream model proposed by Hickok and Poeppel (2007), left IFG and premotor cortex are included in the dorsal language stream, which also includes temporoparietal junction regions. In speech, this stream is assumed to integrate language representations with motor representations, and it is therefore critical for language development. The STS-SRT task involves the forming of linguistic output, and our finding might thus be interpreted as support of the idea that left IFG and sensorimotor regions work together to support integrative language processes. In extension, our finding suggests that the proposed neurocognitive underpinnings of spoken language production might apply also to sign language production. This is not to say that all neurocognitive mechanisms are shared across language modalities (cf., Evans et al., 2019), but as suggested by many before us (e.g., Cardin et al., 2020a; Rönnberg et al., 2021; Trettenbrein et al., 2021), we believe that mechanisms are not unique.

Our results point to that the intrinsic connectivity between left IFG and left sensorimotor regions is sensitive to sign language proficiency and might be stronger in individuals with poor language skills as compared to better skill. Flinker et al. (2015) reported that the left inferior frontal region (Broca’s Area) is typically not co-activated with motor regions in language production. Instead, when activation goes up in motor regions during production, activation in Broca’s Area goes down, and when activation increases in Broca’s Area in pre-articulatory stages, motor regions are relatively silent. As displayed in Figure 2, individuals with stronger sign language sentence reproduction skill tend to have negative intrinsic connectivity between left inferior frontal and sensorimotor regions, whereas individuals with worse skill have positive connectivity between these regions. Thus, intrinsic positive functional connectivity between these regions in deaf adults might be a marker of a language network that is sub-optimally (dis)-integrated, and this might be what we see evidence of in the present study. Proficiency is linked to different developmental trajectories, and the idea that we propose here is thus loosely related to the notion that the dorsal stream is important for language development via input–output matching mechanisms (Hickok and Poeppel, 2007). Sub-optimized network integrity might mean that individuals with weaker proficiency use non-linguistic motor representations to compensate for poorly defined language representations, or that access to language-based motor representations requires greater involvement of language-control functions. Based only on the present study, any definitive conclusion is of course premature. Nevertheless, our results suggest that the strength of intrinsic connectivity between dorsal stream left inferior frontal and sensorimotor regions might be a marker of the level of ability to reproduce sign language sentences.

### Individual Differences in Sign Language Proficiency and Functional Connectivity

Banaszkiewicz et al. (2021) reported a sign language-specific change in task-invoked connectivity between left IFG and left lateral superior occipital cortex in hearing adults who were beginning learners of Polish Sign Language. The task was a sign-based lexical decision task (deciding whether visually presented signs were real or not), which represents an intermediate level of linguistic processing compared to the behavioral measures used in the present study. In our case, we saw a connection that suggests an effect related to effective perception-production processing (the STS-SRT), whereas the results reported by Banaszkiewicz et al. (2021) might reflect improved effectiveness of a perception-identification interface. Another important difference is that Banaszkiewicz et al. (2021) investigated task-based connectivity and not rsFC. Despite the methodological differences between the present study and the study by Banaszkiewicz et al. (2021), both studies suggest that the left IFG might be a critical node in functional networks related to sign language proficiency. How this region is connected functionally and structurally to other regions of the brain should thus be further studied in relation to (sign) language proficiency in future research. To our knowledge, no previous study has investigated task-based functional connectivity in relation to different types of sign language processing skills. Such a study could reveal, by experimental manipulation, what the rsFC-behavior associations revealed in the present study reflect.

In contrast to Li et al. (2013), which was the only previous study on rsFC-behavior associations including deaf participants that we found, we did not see that rsFC within the STG was associated with language proficiency. Although Trettenbrein et al. (2021) noted that the STG might not respond to linguistic sign-based stimuli *per se*, the broader literature indicates that this region is involved in language processing (Hickok and Poeppel, 2007; Friederici, 2012; Bornkessel-Schlesewsky and Schlesewsky, 2013; Cardin et al., 2020a). The lack of statistically significant associations between any of our behavioral measures and connectivity from this region might thus be surprising, although different methodology across studies is a likely explanation. The previous study most like the present study, conducted by Li et al. (2013), reported a correlation between story writing ability and connectivity between right middle superior temporal sulcus and left superior temporal sulcus/STG. Li et al. (2013) did however not observe any statistically significant association between their measure of language skill and rsFC from the superior temporal cortex to other regions of the brain. The lack of similar associations in the present study and the study by Li et al. (2013) might be explained by that the type of language skills investigated were different, or that different seeds were used in the analyses. In the present study, we used the posterior STG language network nodes in Conn as our seeds. This seed is relatively large, and it might therefore be difficult to capture meaningful connectivity in a small sample. On the other hand, using a pre-defined seed makes the design more transparent and facilitates replication. Both we and Li et al. (2013) failed to find that inter-regional rsFC from the STG predicts language skill in deaf adults, and it might be the case that it does not. However, a future study aiming at capturing this might fare better than us by including a larger sample and more precisely delineated seeds.

Since the connectivity observed here is based on rsFC, that is, co-activation between brain regions when the participants have no specific task to perform, the associations between regions are not invoked by a language task and therefore might not have anything specific to do with language processing. As mentioned earlier in the Discussion, one risk with a small sample is that findings are random. We tried to minimize the risk for this by restricting the number of statistical tests we performed. However, we also wanted to maximize statistical power and therefore applied a liberal approach when correcting for multiple statistical tests. Since the one association that we saw fits reasonably well with the existing literature, we believe that it is a meaningful association. At the same time, it does probably not represent the only relevant association. Based on previous empirical findings (Trettenbrein et al., 2021) and theoretical considerations (Hickok and Poeppel, 2007), we selected four language-relevant nodes as our seeds, but several potentially relevant regions were not included (e.g., supramarginal gyrus, anterior temporal regions, lateral occipital cortex). Another limitation is that we were only able to detect effects that were strong. This is reflected in the effect sizes of observed statistically significant effects, ranging from *R*^2^ 0.75 to 0.87. Thus, in addition to the risk of finding random association, the small sample we included also carries a risk of missing out on meaningful effects that are small in magnitude.

Our results indicated an association between only one of the behavioral measures and rsFC. This might be because the measures tap onto different aspects of linguistic processing, or that task demands differ. We cannot determine whether the results we see are driven by a specific linguistic skill, or by any other skill or task-dependent factor. C-PhAT is performed by mentally converting orthographic input to sign-based representations, and then comparing representations before a decision is made explicit by a timed button press. In the STS-SRT task, on the other hand, the participant views and repeats a sign language sentence, with no further decision to make. The STS-SRT demands that a sentence is produced and the C-PhAT includes covert production of signs, as the individual mentally represents handshapes and compares them. Additionally, the STS-SRT includes phonological, lexical, and syntactical knowledge-structures, combined into a coherent expression, whereas C-PhAT taxes isolated phonological processing ability, and, given its design, orthographic-phonological mapping. On top of this, the STS-SRT has a social component (i.e., viewing another person producing a sentence) that the C-PhAT does not. Taken together, it is difficult to identify a specific origin of differences in associations observed between these tasks. We saw that better performance on C-PhAT had a positive association with non-verbal cognitive ability, and we controlled for scores on the Visual Puzzles task in connectivity analysis on C-PhAT. Thus, the influence of general cognitive factors in the association for that task was controlled for. We did not make the same control for STS-SRT, since no association with non-verbal cognitive ability was observed and adding a covariate to the analysis would then only reduce statistical power. However, non-linguistic processing skills might explain the observed relationships and differences in associations between tasks in the present study. In summary, our study has some methodological issues that future studies should correct, including the small sample size and the selection of seeds. In addition, future work should also carefully consider which behavioral measures to include. However, given that the studied population is a unique group and our approach here is novel we believe that the present findings represent an important contribution to the field.

Both structural and functional plasticity as a result of deafness and sign language use has been reported in the literature (Sadato et al., 2004; Cardin et al., 2013, 2018; Olulade et al., 2014; Ding et al., 2015; Twomey et al., 2017; Benetti et al., 2018; Trumpp and Kiefer, 2018; Bonna et al., 2020; Finkl et al., 2020; Andin et al., 2021; Dell Ducas et al., 2021; for reviews see Alencar et al., 2019; Cardin et al., 2020b). Thus, brain connectivity patterns that underlie linguistic operations in deaf sign language users might not be the same as for hearing individuals who use speech (or sign language). In our design, we did not compare across groups and previous studies on hearing individuals with a similar design as the present study are lacking. Although the general pattern of the available studies is that there is a great deal of overlap in the neural underpinnings of language processing regardless of modality (Walenski et al., 2019; Cardin et al., 2020a; Trettenbrein et al., 2021), a few regions might be modality-specific (Trettenbrein et al., 2021) and differences might exist at a form-based representational level (Evans et al., 2019). In addition, the type of linguistic operation might interact with modality effects. Studies with a design that allows for comparisons across language modality and different types of linguistic tasks are well needed to improve our understanding of how language processing is represented neurally, both in terms of modality–specificity and modality–generality.

## Conclusion

Intrinsic functional connectivity between inferior frontal and sensorimotor cortical regions is associated with accurate sign language reproduction. This suggests that the cortical interaction at rest between dorsal language stream regions might be a marker of sign language proficiency, and more specifically the ability to reproduce sign language. Development of sign language skill might be determined by brain connectivity, or language development might form the connectivity between brain regions.

## Data Availability Statement

Data is provided by the first author upon request.

## Ethics Statement

The studies involving human participants were reviewed and approved by the Regional Ethical Review Board in Linköping (Dnr 2016/344-31). The participants provided their written informed consent to participate in this study.

## Author Contributions

EH and JA conceptualized and designed the study, collected data, and performed data analysis. KS scored performance on the STS-SRT. All authors were involved in the interpretation of results as well as preparing and finalizing the manuscript after a draft was prepared by EH.

## Conflict of Interest

The authors declare that the research was conducted in the absence of any commercial or financial relationships that could be construed as a potential conflict of interest.

## Publisher’s Note

All claims expressed in this article are solely those of the authors and do not necessarily represent those of their affiliated organizations, or those of the publisher, the editors and the reviewers. Any product that may be evaluated in this article, or claim that may be made by its manufacturer, is not guaranteed or endorsed by the publisher.

## Footnotes

1
www.nitric.org/projects/conn
